# Cement-Based Piezoelectric Ceramic Composites for Sensing Elements: A Comprehensive State-of-the-Art Review

**DOI:** 10.3390/s21093230

**Published:** 2021-05-07

**Authors:** Weijian Ding, Yuqing Liu, Tomoki Shiotani, Quan Wang, Ningxu Han, Feng Xing

**Affiliations:** 1 Department of Civil and Earth Resources Engineering, Graduate School of Engineering, Kyoto University, Kyoto 615-8540, Japan; dingweijian1992@foxmail.com (W.D.); shiotani.tomoki.2v@kyoto-u.ac.jp (T.S.); 2 Department of Mechanics and Aerospace Engineering, Southern University of Science and Technology, Shenzhen 518055, China; wangq@sustc.edu.cn; 3 Guangdong Province Key Laboratory of Durability for Marine Civil Engineering, College of Civil and Transportation Engineering, Shenzhen University, Shenzhen 518060, China; nxhan@szu.edu.cn (N.H.); xingf@szu.edu.cn (F.X.)

**Keywords:** sensing element, piezoelectric ceramic composite, fabrication, properties, structural health monitoring

## Abstract

Compatibility, a critical issue between sensing material and host structure, significantly influences the detecting performance (e.g., sensitive, signal-to-noise ratio) of the embedded sensor. To address this issue in concrete-based infrastructural health monitoring, cement-based piezoelectric composites (piezoelectric ceramic particles as a function phase and cementitious materials as a matrix) have attracted continuous attention in the past two decades, dramatically exhibiting superior durability, sensitivity, and compatibility. This review paper performs a synthetical overview of recent advances in theoretical analysis, characterization and simulation, materials selection, the fabrication process, and application of the cement-based piezoelectric composites. The critical issues of each part are also presented. The influencing factors of the materials and fabrication process on the final performance of composites are further discussed. Meanwhile, the application of the composite as a sensing element for various monitoring techniques is summarized. Further study on the experiment and simulation, materials, fabrication technique, and application are also pointed out purposefully.

## 1. Introduction

Infrastructure, a series of fundamental facilities and structure systems, performs indispensable support for society, expected to achieve sustainability and economic efficiency. However, safety hazards caused by progressive deterioration with age [1] and serious disasters related to severe environmental conditions (freeze–thaw cycles [2,3], marine environment [4], high temperature [5], etc.) are the potential issues during the lifespan of infrastructure, possibly leading to a shortened service life and high maintenance/reconstruction costs. Concrete, regarded as an affordable, durable, and dramatic building material, has been widely applied in infrastructure [6,7,8,9,10]. Due to the adverse impact of intrinsic (material self-defects [11], deficient structural design and construction, etc.) and extrinsic (severe environment, accidental loading [12], etc.) factors, concrete structures usually undergo deterioration during the whole lifespan, such as concrete cracking [13], steel corrosion [14,15], spalling [16], and structure collapse [17].

Most serious durability and safety issues for concrete-based infrastructure are usually the cumulative consequence induced by the service environment [18,19], aging [20], and self-defects [21]. At different periods of its lifespan, there are various dominant factors for degeneration. In the concreting process, fresh concrete can be easily influenced by temperature, humidity, and rheological properties, possibly leading to self-defects (cracks and pores). During the service life, aggressive action related to the invasive substances (chloride, carbon dioxide, and sulfate, etc.) and environmental change is the major reason for the deterioration (e.g., corrosion, carbonation, and cracking). As concrete structures age, the deterioration will decrease the ultimate load capacity and further bring safety and serviceability risks. The application of eco-friendly materials (e.g., recycled concrete [22] and aggregate [10], seawater [23] and sea sand [24], and geopolymer [25,26]) in construction is another challenge for the structural performance. Due to durability and safety issues, concrete-based infrastructure will struggle to maintain functionality, and the most affordable solution (e.g., repair, upgrade, and partial reconstruction) needs to be filtrated.

Considering the severe consequences caused by structural deterioration, there is a strong demand for implementing identification strategies and protection for concrete-based infrastructure. An innovative, reliable, and cost-effective structural health monitoring (SHM) technique for constructing/existing infrastructure has been an essential item to diagnose and mitigate damages and further ensure its functionality, thereby elongating the service life. The strategy of efficient and accurate SHM systems with intelligent materials (e.g., optical fiber [27], piezoelectric materials [28]) installed in concrete structures has attracted lots of attention in recent decades [29,30,31,32]. Piezoelectric materials can be encapsulated as smart aggregates and embedded into the concrete structure, thereby monitoring the deterioration process. The distinction in acoustic impedance, density, and mechanics properties leads to the lower compatibility between the sensing element and host structures, resulting in signal capture disturbance. Table 1 summarizes the major parameters that lead to the incompatibility among cement, concrete and piezoelectric ceramics. One of the critical factors for signal acquisition is acoustic impedance, determined by the density and acoustic velocity [33]. The acoustic velocities of piezoelectric ceramic, cement, and plain concrete are similar, while the density of piezoelectric ceramic is much higher, resulting in the acoustic impedance mismatching problem. The piezoelectric composite is an alternative approach to address this issue. In 2002, cement-based piezoelectric composites (CPCs), innovated by Li et al. [34], are regarded as a pioneering inorganic piezoelectric composite, more adaptable to the concrete structure. Based on the connectivity, piezoelectric composite materials can be divided into 10 basic types [35]. The superiority of the 0-3/1-3/2-2 type CPCs applied in SHM has been demonstrated. The 1-3 [36]/2-2 [37] types can be regarded as the development based on 0-3 by controlling the distribution of piezoelectric materials in the cement matrix. Despite the lower piezoelectric strain constant (*d*_33_), 0-3 CPCs show an excellent overall performance (e.g., higher piezoelectric voltage constant (*g*_33_), acoustic impedance matching, and flexibility) as a better alternative material for sensing elements, and also show a prospect in combining intelligent manufacturing. However, the difference in fabrication parameters and various raw materials sources leads to a great variety in the final piezoelectric performance.

In the last two decades, the performance improvement of CPCs has been performed. The significant variety in the final piezoelectric performance illustrates the existing shortcoming and insufficient understanding around the composite. Our main objective in this review is to recapitulate the previous studies related to CPCs and discuss the influence of raw materials and the main problems in fabrication, thereby promoting advanced piezoelectric composite design, fabrication, and application. Moreover, the present review will summarize previous research to sort out the critical influencing factors and potential directions. Then, to clarify the influencing factors of the composite materials, theories and fundamentals, experimental and modeling analysis, raw materials, the fabrication process, and application are presented in different sections. At the end of each section, insightful viewpoints and prospective studies on the composite will be provided.

## 2. Recent Study on Cement-Based Piezoelectric Composites

The piezoelectric performance (e.g., piezoelectric, electromechanical coupling, and dielectric properties) of CPCs has been comprehensively characterized and discussed. Initially, Li et al. [30] reported the feasibility of CPCs and characterized their performance, including the piezoelectric strain factor (*d*_33_), piezoelectric voltage factor (*g*_33_), electromechanical coupling coefficient (*K_t_* and *K_p_*), and dielectric constant (*ε_r_*). Subsequently, Huang et al. [46] studied the polarization process of PZT/sulphoaluminate cement composites; Chaipanich et al. [47] demonstrated the properties of PZT–ordinary Portland cement composites. These studies have demonstrated the feasibility of CPCs and revealed that the piezoelectric particles’ higher content could improve the piezoelectric performance. Additionally, Chaipanich et al. [48] studied the particle size effect, showing that a larger particle of the function phase is beneficial for improving the *d*_33_ and *ε_r_*. Pan et al. [49] and Chomyen et al. [50] demonstrated improved piezoelectric performance by adding fly ash, respectively. However, the final piezoelectric performance shows a tremendous difference, mainly attributed to the difference in piezoelectric materials and polarization parameters. According to the above researches, the main influencing factors on the all-round performance can be summarized as the (a) matrix; (b) functional phase (piezoelectric materials); (c) fabrication process; (d) aging.

Due to the complex hydration product composition and heterogeneous microstructure, the matrix effect on piezoelectric performance has been investigated. Among these, enhancing *d*_33_ is still the primary target. The low-efficiency stress transfer between matrix and piezoelectric particles caused by the poor connectivity and porosity is the main reason for the lower *d*_33_. Therefore, a denser matrix is a penitential approach to optimize it. Chaipanich et al. [51] revealed that the *d*_33_ shows a slight increase with adding silica fume. Wang et al. [52] found that adding silica-based material can improve the *d*_33_ even up to 99.0 pC/N due to the ITZ optimization under the conditions of compression forming, steam curing, and aging. Subsequently, to decrease porosities, slag, fly ash, and kaolin was studied by Pan et al. [49,53], tracing the piezoelectric properties in different ages, and the highest *d*_33_ can reach 111.1 pC/N. Wittinanon et al. [38] employed PVDF to modify the ITZ and reduce the porosity, showing a significant optimization in reducing leakage current. Considering the positive effect of the higher matrix conductivity during the polarization process, carbon materials (carbon addition [54], carbon black [55,56], carbon nanotube [57], and graphene nanoplatelets [58]) were also applied to optimize the piezoelectric properties. The matrix properties can also affect the *g*_33_, *K_t_*, *K_p_*, *ε_r_*, and dielectric loss (*tan δ*), and the increase of parameter values (*K_t_*, *ε_r_*, and *g*_33_) with the help of admixture has been reported [49,55]. The output voltage (*V*) of the composite mixed with basalt fiber, which affects the matrix’s elastic modulus, was also characterized [43].

Meanwhile, the fabrication process optimization was also carried out, and some steps demonstrate significant improvement. Huang et al. [59] applied the forming pressure to fabricate the CPCs, which could help obtain a denser matrix and further enhance the *d*_33_. Furthermore, the curing is also essential to obtain the higher piezoelectric performance because inadequate curing would cause interfacial cracks and lead to a locally poor value of *d*_33_. Considering the positive effect of the high temperature on the hydration evaluation, Wang et al. [52] carried out the hot water and steam curing process, respectively. Pan et al. [60] found that the pre-heating treatment could improve the polarization efficiency due to decreased moisture. The above fabrication process development could contribute to a better microstructure and mitigate the negative effect caused by the ITZ between the cement matrix and piezoelectric ceramic particles. The significance of polarization has been studied by Huang et al. [46] and Dong et al. [61], demonstrating that the voltage, polarization time, and temperature can directly play a decisive role.

Recently, the multi-factors coupling for the design and fabrication of the composite has been considered. Among those factors, aging is the key influential factor combined with the materials and fabrication process for the performance of CPCs. Dong et al. [37] and Huang et al. [62] revealed the *d*_33_ increase with time, even though the matrix phase in their studies is different. Later, Chaipanich et al. [63] found the increased trend of *d*_33_ in PZT-Portland cement composites with time; Pan et al. [53] also characterized this phenomenon during their investigation into the effect of admixture. In 2016, Pan et al. [60] found that heat treatment could improve the comprehensive performance after aging. Subsequently, the effect of the water/cement ratio and time on the piezoelectric performance was also studied [64].

The application of this composite has been carried out. Lu et al. [29,30,40,65] systemically monitored the different states in concrete using embedded CPCs sensors, including hydration, crack, and corrosion. Xing et al. [66] tested the electrical response of this material under different mechanical loadings. Pan et al. [31] applied the composite for monitoring the strength growth of concrete via electromechanical impedance. Those applications reveal the feasibility and superiority of CPCs as a potential sensing element.

Regarding the environmental issues, lead-free piezoelectric material has attracted increasing attention. Rianyoi et al. [38,67,68,69] prepared the barium titanate-cement composites and characterized the influence of the particle size and polyvinylidene fluoride (PVDF). Chaipanich et al. [39,50,70,71,72,73] fabricated barium zirconate titanate-cement composites and studied their microstructure and piezoelectric performance. Hunpratub et al. [74] believe that BCTZO (Ba_0.85_Ca_0.15_Ti_0.9_Zr_0.1_O_3_) particle is an alternative material as function phase and revealed the effect of particle size on dielectric and piezoelectric properties. Additionally, BNBT (0.94(Bi_0.5_Na_0.5_)TiO_3_-0.06BaTiO_3_) [75] and BNBK (0.88Bi_0.5_Na_0.5_TiO_3_-0.08Bi_0.5_K_0.5_TiO_3_-0.04BaTiO_3_) [76] have been used as a function phase to fabricate the composite. Although lead-free piezoelectric materials are potential functional materials, the lower piezoelectric properties and poor temperature stability still limit their application in CPCs.

**Figure 1:**
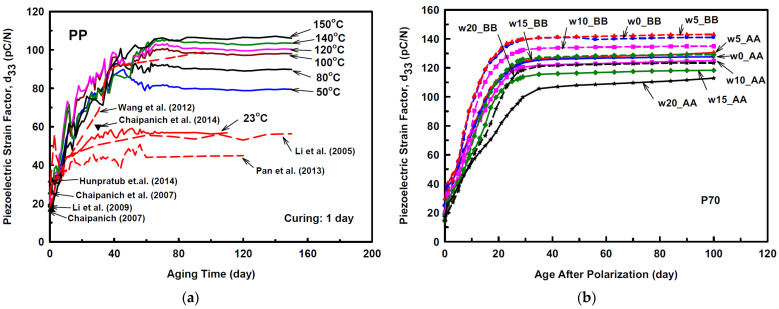
(**a**) Piezoelectric strain constant of cement piezoelectric composites. Reprinted with permission from ref. [83]. Copyright 2016 Elsevier. (**b**) Piezoelectric strain factor of 70% PZT/cement composites versus age. Reprinted with permission from ref. [64]. Copyright 2013 Elsevier.

Numerous studies have illustrated the feasibility to fabricate and employ this composite as a sensing element. However, the effect of the fabrication process and polarization parameters are still essential to further study due to the physical and chemical distinction between cementitious materials and piezoelectric materials. The properties of lead-based/lead-free piezoelectric ceramic and its cement-based composites in recent studies, including *d*_33_, *g*_33_, *ε_r_, K_t_*, and acoustic impedance, are intuitively summarized in Table 2. The highest value of *d*_33_ in lead-free CPCs is 61.5 pC/N, while that of lead-bearing CPCs is 87 pC/N initially. However, the *d*_33_ of the lead-bearing composite can reach over 140 pC/N after aging, close to the piezoelectric ceramic. It should be mentioned that these higher parameters almost attribute to the positive effect of aging, and the typical studies for tracing the change of *d*_33_ with aging are illustrated in Figure 1. Only a few studies can obtain high piezoelectric performance at the initial stage after polarization. Santos et al. [77] reported that the curing process of CPCs has a direct relationship with its dielectric properties and electrical conductivity, attributing to the existence of unstable dipoles, which would be a suitable example for understanding the performance variation, illustrating the significant effect of the matrix properties on the final performance. Kantakam et al. [78] revealed the tremendous influence of the matrix material on the dielectric properties.

## 3. Theory, Experiment and Simulation

### 3.1. Fundamentals of Cement-Based Piezoelectric Composites

Rigorous theoretical analysis can describe the approach to achieve acoustic impedance matching, aiming to reduce signal reflection and loss. For the concrete-based structural health monitoring, there is a non-negligible interface between the host structure and sensor during the elastic wave propagation process [33]. The interface will lead to partial signal energy loss. The CPCs have been developed to reduce the interface influence and obtain high-quality signals. The basic properties of host materials and piezoelectric composite are needed to accomplish the impedance matching, including the density, volume percentages, and elastic moduli. Subsequently, the density and elastic moduli of composites can be calculated by Equations (1) and (2), respectively [34]:

(1) $$\rho_{c}=\rho_{1}v_{1}+\rho_{2}v_{2}$$

(2) $$E_{c}=\frac{1}{v_{1}/E_{1}+v_{2}/E_{2}},$$

where *ρ* and *E* represent the density and elastic moduli, respectively; subscript *c*, 1, and 2 stands for the composite, ceramic particle, and cement matrix, respectively; ν represents the volume percentage.

Then, the acoustic velocity (*V_c_*) and acoustic impedance (*Z_c_*) of CPCs can be expressed as Equations (3) and (4), respectively.

(3) Vc=(Ecρc)12,

(4) Zc=ρcVc=(ρcEc)12,

Thus, the acoustic impedance of CPCs can be adjusted based on Equation (4), thereby achieving acoustic impedance matching.

In terms of function, the piezoelectric effect is the source of the mechanical–electrical conversion of piezoelectric materials related to the asymmetric crystal structure. It can be divided into direct and reverse piezoelectric effects. The direct piezoelectric effect, an essential basis of the piezoelectric sensor, describes the free electric charges on the crystal surface induced by mechanical force; the reverse piezoelectric effect refers to the mechanical deformation of the crystal caused by the external electric field, also known as the electrostrictive effect. Since discovering the piezoelectric effect in quartz crystals in the 1980s, the fundamentals for piezoelectric materials have been deeply studied.

The stress (*T*) and electric field (*E*) can both directly induce electric displacement (*D*) for piezoelectric materials. The *D* directly aroused by the *T* is [84]:

(5) D=dT,

where *d* present the piezoelectric strain constant. Under the internal electric field (*E*) without external stress, *D* can be given by:

(6) $$D=\varepsilon^{T}E,$$

where *ε^T^* presents the dielectric permittivity for constant stress. Moreover, the strain of the piezoelectric materials can be caused by *T* and internal *E*, respectively.

*S = s^E^T*, (7)

*S = dE*, (8)

where *s^E^* is the elastic compliance for the constant electric field.

Furthermore, the coupling between dielectric and elastic properties of the piezoelectric materials can be described by a linear relationship between two electrical (*E* and *D*) and mechanical (*T* and *S*) variables, illustrating the direct and reverse piezoelectric effects, and the state equations are:

*D = dT + ε^T^E*, (9)

*S = s^E^T + dE*, (10)

when *S* and *E* are selected as variables, the state equation of the piezoelectric effect can be written as [66]:

*T* = *c^E^S* − *e_m_E*, (11)

*D = e_n_S + ε^S^E*, (12)

where *c^E^* represents the elastic stiffness coefficient with a constant electrical field; *e_m_* and *e_n_* represent the piezoelectric stress coefficient, respectively; *ε^S^* denotes the dielectric coefficient with constant strain. Equations (11) and (12) are regarded as more convenient equations to describe the piezoelectric effect of ferroelectric crystal, which have been applied to study CPCs under the mechanical loadings condition [66].

**Figure 2:**
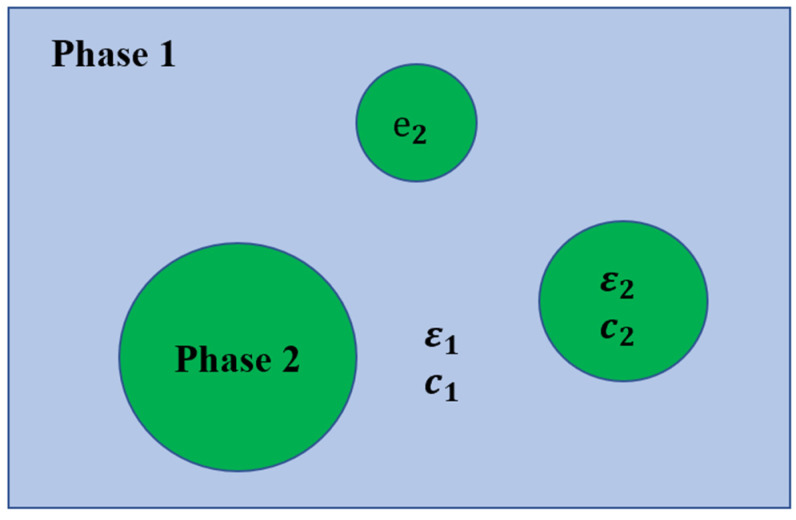
Two-phase system composed of a non-piezoelectric continuous phase (Phase 1) and a piezoelectric spherical phase (Phase 2). Reprinted with permission from ref. [87]. Copyright 1973 John Wiley & Sons, Inc.

A clear description of the mechanical–electrical conversion relationship between the matrix and piezoelectric particles is vital in the piezoelectric composite. For the CPCs, the external stress is acting on the non-piezoelectric matrix instead of piezoelectric particles, and the direct piezoelectric effect is relative to the volume percentage of piezoelectric materials. A two-phase system (as depicted in Figure 2) which has been employed to describe the epoxy/PZT composite systems, can be used to describe the piezoelectric effect of cement/PZT composites. Based on the elastic constant ($c_{1\;}\mathrm{and}\;c_{2}$) and dielectric constant ($\varepsilon_{1\;}$and $\varepsilon_{2}$) of two phases and the volume percentage of Phase 2(∅), the apparent elastic constant c and dielectric constant ε of the two-phase system are shown as below:

(13) $$c=\frac{3\left(1-\emptyset \right)c_{1}+\left(2+3\emptyset \right)c_{2}}{\left(3+2\emptyset \right)c_{1}+2\left(1-\emptyset \right)c_{2}}c_{1},$$

(14) $$\varepsilon =\frac{2\left(1-\emptyset \right)\varepsilon_{1}+\left(1+2\emptyset \right)\varepsilon_{2}}{\left(2+\emptyset \right)\varepsilon_{1}+\left(1-\emptyset \right)\varepsilon_{2}}\varepsilon_{1},$$

**Figure 3:**
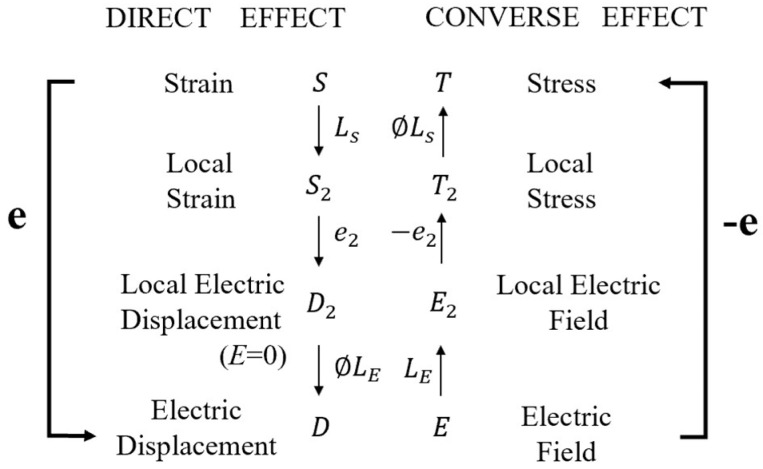
Piezoelectric effect for a two-phase system. Reprinted with permission from ref. [87]. Copyright 1973 John Wiley & Sons, Inc.

These two constants can help to understand the relationship between electric displacement and strain. Figure 3 elucidates the piezoelectric effect of the two-phase system, different from that of piezoelectric ceramics. The direct piezoelectric effect can be written as:

(15) D=eS,

However, Phase 1 is the non-piezoelectric matrix without the piezoelectric effect. The only approach to induce the direct piezoelectric effect is applying stress to Phase 2, the piezoelectric spherical phase. To elucidate the direct piezoelectric effect, a piezoelectric constant e is defined:

(16) $$e={\left(\frac{D}{S}\right)}_{E}=-{\left(\frac{T}{E}\right)}_{s},$$

The strain acting on the two-phase system will produce the local strain in Phase 2 due to the strain induced by external loading in Phase 1.

(17) $$S_{2}=L_{s}S,$$

where $S_{2}$ is the local strain in Phase 2, $L_{s}$ is the local field coefficients with respect to strain (S).

Subsequently, the local electric displacement ($D_{2}$) is aroused by $S_{2}$.

(18) $$D_{2}=e_{2}S_{2},$$

where $e_{2}$ represents the piezoelectric constant of Phase 2, and the relationship between e and $e_{2}\;$can be expressed as:

(19) $$e=\emptyset L_{s}L_{E}e_{2},$$

where $L_{s}$ and $L_{E}$ represent the local field coefficients with reference to S and E, respectively.

In the condition of E = 0, the apparent electric displacement D aroused by $D_{2}$ can be written as:

(20) $$D=\emptyset L_{E}D_{2},$$

The local field coefficients$\;L_{s}\;$and $L_{E}$ are calculated based on Equations (13) and (14) [85,86,87,88]:

(21) $$L_{s}=\frac{5c_{1}}{\left(3+2\emptyset \right)c_{1}+2\left(1-\emptyset \right)c_{2}},$$

(22) $$L_{E}=\frac{3\varepsilon_{1}}{\left(2+\emptyset \right)\varepsilon_{1}+\left(1-\emptyset \right)\varepsilon_{2}},$$

Therefore, the local strain and field coefficients are the major factors affecting the piezoelectric performance, which relate to the dielectric constant of two phases and the volume percentage.

To predict the piezoelectric properties of the composite, the cube, parallel and series theoretical models have been employed [34,69,88]. The results [34,61,69,88,89] reveal that the cube model is more suitable for piezoelectric properties prediction of 0-3 cement-based piezoelectric ceramic composite. For the dielectric constant, the theoretical predictions according to different models are calculated as follows [69,88]:

(23) $$\frac{1}{\varepsilon }=\frac{v_{1}}{\varepsilon_{1}}+\frac{v_{2}}{\varepsilon_{2}},\;(\mathrm{series}\;\mathrm{model})\;$$

(24) $$\varepsilon =\varepsilon_{1}\cdot v_{1}+\varepsilon_{2}\cdot v_{2},\;\left(\mathrm{parallel}\;\mathrm{model}\right)$$

(25) $$\varepsilon =\frac{\varepsilon_{1}\cdot \varepsilon_{2}}{(\varepsilon_{2}-\varepsilon_{1})\cdot v_{1}^{-1/3}+\varepsilon_{1}\cdot v_{1}^{-2/3}\;}+\varepsilon_{2}\cdot \left(1-v_{1}^{2/3}\right),\;(\mathrm{cube}\;\mathrm{model})\;$$

The theoretical models for piezoelectric strain factor are [89,90]:

(26) $$d_{33}=\frac{v_{1}\cdot d_{33}^{1}\cdot \varepsilon_{2}+v_{2}\cdot d_{33}^{2}\cdot \varepsilon_{1}\;}{\varepsilon_{2}\cdot v_{1}+\varepsilon_{1}\cdot v_{2}},\;(\mathrm{series}\;\mathrm{model})\;$$

(27) $$d_{33}=\frac{v_{1}\cdot d_{33}^{1}\cdot S_{33}^{2}+v_{2}\cdot d_{33}^{2}\cdot S_{33}^{1}\;}{S_{33}^{2}\cdot v_{1}+S_{33}^{1}\cdot v_{2}},\;\left(\mathrm{parallel}\;\mathrm{model}\right)$$

(28) $$d_{33}=d_{33}^{2}\;\cdot \;\frac{v_{2}}{v_{2}^{1/3}+\left(1-v_{2}^{1/3}\right)\cdot \;\frac{\varepsilon_{2}}{\varepsilon_{1}}}\;\cdot \;\frac{1}{1-v_{2}^{1/3}+v_{2}},\;\left(\mathrm{cube}\;\mathrm{model}\right)$$

where $d_{33}^{},\;v,$ and $S_{33}^{}$ represent the piezoelectric strain factor, volume percentage, and elastic compliance, respectively; super-/subscript 1 and 2 stand for the cement matrix phase and ceramic phase, respectively.

### 3.2. Performance and Microstructure Characterization

As an alternative functional material for sensing elements, it is essential to characterize the piezoelectric performance. The piezoelectric strain factor (*d*_33_) and piezoelectric voltage factor (*g*_33_) of CPCs related to the macroscopic physical quantity are the basic parameters, which characterize the coupling relationship between the elastic and electrical polarization effects of piezoelectric bulk. Dielectric constant (*ε_r_*) and dielectric loss (*tan δ*) are the other factors to evaluate the convenience of polarization. The impedance spectrum is used to obtain electromechanical coupling coefficients (*K_t_* and *K_p_*) and further characterize the mechanical–electrical conversion efficiency of the piezoelectric composites. The mechanical quality factor (*Q_m_*), describing the ability to overcome the energy consumed by internal friction in the piezoelectric resonance state, is another critical parameter. Recently, lead-free piezoelectric materials have been employed as function phases in CPCs, such as BT, BZT, and BCTZO. Chaipanich et al. [68,71] employed the polarization-electric field loops (P-E loops) to characterize the ferroelectric hysteresis properties of BT/BZT cement composites.

Considering the coupling complexity between the cement matrix and ceramics particles, a microstructural analysis of the composites was conducted to evaluate the optimization process. Jaitanong et al. [91] employed piezo-response force microscopy to investigate the microstructure of the composite after hydration, thereby characterizing the local piezoelectricity of the domains of PZT particles. Potong et al. [72] considered the effect of the complex hydration condition and the various hydration products in the cementitious material and applied linear voltage-differential transformer dilatometer to test the thermal expansion, indicating that the thermal expansion the coefficient of the composites with 30~70% BZT ceramic powder is similar to that of concrete. Wittinanon et al. [39] evaluated the effect of PVDF on the composite’s mechanical properties using the indentation technique. Moreover, acoustic impedance, porosity, and leakage current of the composite with different PVDF content were also characterized [38].

### 3.3. Modelling Analysis

**Figure 4:**
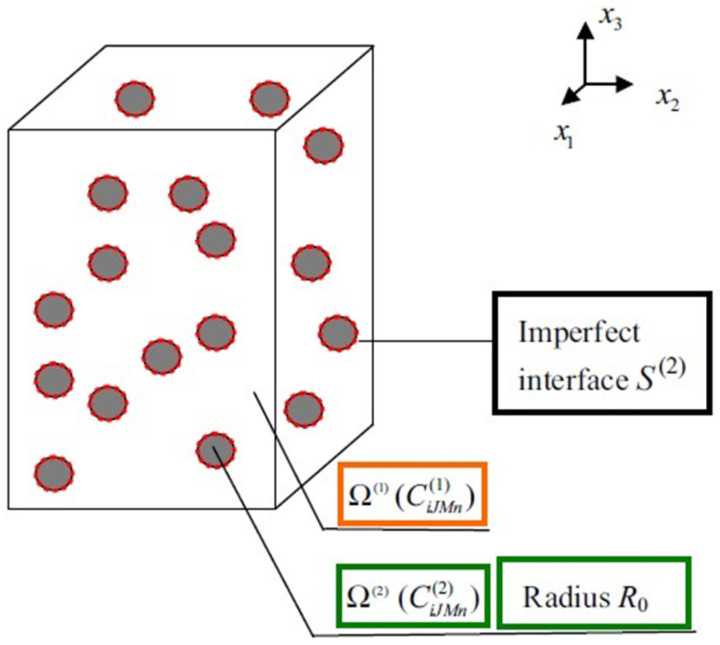
Schematic illustration for composite materials with imperfect interface. Reprinted with permission from ref. [92]. Copyright 2016 IEEE.

**Figure 5:**
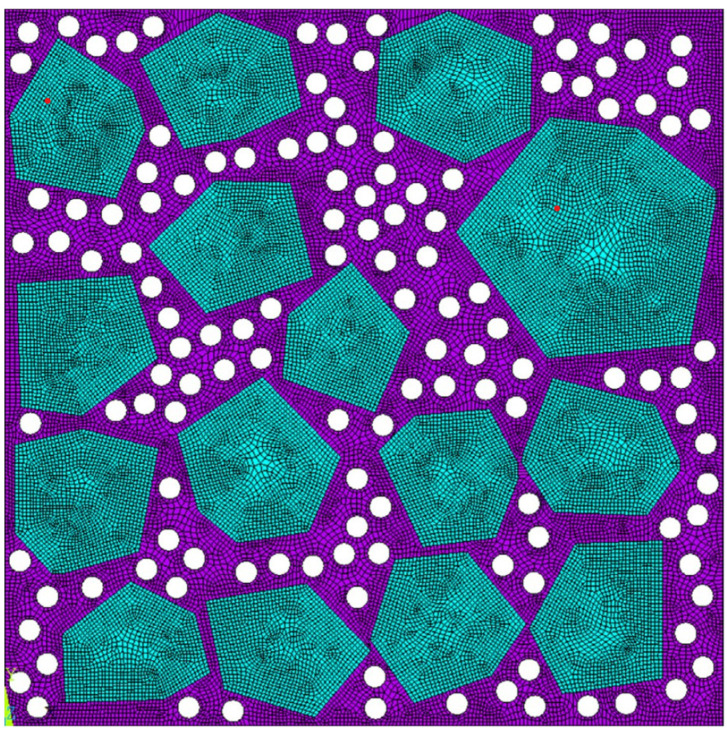
Finite element mesh of the porous PZT composite with the largest volume fraction. Reprinted with permission from ref. [93]. Copyright 2006 Elsevier.

To understand the theoretical modeling, the size-dependent phenomenon of the CPCs initially attracted attention. Wang et al. [92] consider the minor defect in the interface (Figure 4) and proposed a modified micromechanics model to obtain the effective moduli of particle-reinforced piezoelectric composites. The size effect, effective piezoelectric strain factor ($d_{33}^{Eff}$), and relative dielectric constant ($\frac{k_{33}^{\sigma -Eff}}{k_{0}}$) are further discussed. Calculated results revealed a similar increasing trend of both $d_{33}^{Eff}$ and $\frac{k_{33}^{\sigma -Eff}}{k_{0}}$ with the increase of piezoelectric particle size. Meanwhile, the calculating and experimental results were in good agreement, indicating that the micro-scale size effect exists. Attempting to investigate the effect of pores and piezoelectric particles, Sladek et al. [93] investigated the effective thermo-electro-mechanical material properties of CPCs based on the micromechanics’ representative volume element (Figure 5). The results illustrated the effective elastic, and the piezoelectric coefficients are enhanced with the increase of piezoelectric particle volume, while the effective heat and conduction coefficients are decreased. Additionally, the increase of porosity will result in the effective elastic, piezoelectric, and heat conduction coefficients decreasing.

### 3.4. Future Study

Based on the fundamentals and two-phase system, the complexity of the mechanical–electrical conversion process in the composite has been illustrated. Meanwhile, the local strain and field coefficients related to how the non-piezoelectric continuous phase’s mechanical properties can dramatically affect the direct piezoelectric effect need further study. Experimental studies on the microstructure of the composite [52,62] have revealed the existence of defects in the ITZ between the ceramic particles and matrix due to insufficient hydration, resulting in poor local mechanical properties and lower piezoelectric properties. With the employment of finite element methods, the research approach for piezoelectric composite materials can be expanded. However, the complex microstructure of composites attributed to the material distinction and insufficient understanding of the mechanical properties and microstructure after polarization may limit its simulation development. The intense environment (higher voltage and temperature) during the polarization will cause microstructural changes in cement products [94,95], and some damages due to the chemical decomposition will lead to a decrease in mechanical properties. The mechanical and piezoelectric properties of the composite should be equally focused. The mechanical properties of ITZ can dramatically affect the mechanical–electric response due to the stress buffer between the piezoelectric ceramic particles and cement matrix. Further study on the mechanical parameters (e.g., elastic modulus and Poisson’s ratio) of the composite after polarization can promote the simulation development and understanding of its mechanism.

## 4. Materials for Cement-Based Piezoelectric Composites

In view of promoting the design and fabrication development of CPCs, it is essential to understand the properties of the three different components: (i) function phase materials (piezoelectric materials), (ii) matrix phase materials (cementitious materials), and (iii) enhanced phase materials (admixtures). The existence of the distinction in those raw materials and the chemical reactions make the microstructure and chemical properties more complex. For example, Dong et al. [96] reported the chemical reactions at the ITZ between the piezoelectric particles and cement matrix, believing that there exists a coupling effect. Wang et al. [52] found that the silicon-based materials can help obtain denser microstructure, thereby obtaining higher performance of the composite, and Cheng et al. [62] believe that a higher hydration degree can obtain better piezoelectric performance. In this section, reviewing the basic materials is conducted. By summarizing the considerations of related materials, alternative materials for fabricating potential CPCs with better performance are constructively provided, and the future study on materials for CPCs is also discussed.

### 4.1. Piezoelectric Ceramic for CPCs

Piezoelectric materials can be divided into three categories [34]: piezoelectric polymers, piezoelectric composites, and piezoelectric ceramics. Piezoelectric ceramics have been well-studied based on the solid solutions of lead-bearing materials (e.g., PbZrO_3_ (PZ), PbTiO_3_ (PT)), and lead-free materials (e.g., BaTiO_3_ (BT)). Piezoelectric ceramic powder for fabricating the CPCs are all ferroelectric materials, such as lead zirconate titanate (PbTi_n_Zr_1-n_O_3_; PZT) [48], barium titanate (BaTiO_3_; BT) [67], lead magnesium niobate (xPb(Mg_n_Nb_1-n_)O_3_-yPbTiO_3_-zPbZrO_3_; PMN) [62], lead lithium niobate (xPb(Li_n_Nb_1-n_)O_3_-yPbTiO_3_-zPbZrO_3_; PLN) and barium zirconate titanate (Ba(Zr_n_Ti_1−n_)O_3_; BZT). All of them have perovskite-type structures, with the chemical formula ABO_3_. The most used piezoelectric material in SHM is PZT, which can be divided into soft and hard types. Due to higher domain mobility leading to high sensitivity, soft PZT is regarded as an excellent piezoelectric material for the sensing element. The special properties of the soft PZT include the higher *d*_33_ and coupling factors. Generally, lead-bearing piezoelectric materials are produced under high temperatures (about 800~1300 °C) [63], which easily causes lead volatilization and further results in the environmental problem. Due to eco-friendliness issues, lead-free piezoelectric materials have received increasing attention to fabricating the piezoelectric composites, such as BT, BZT, BCTZO, BNBK, and BNBT.

As the function phase, piezoelectric materials are the particle covered by the matrix, directly endowing the composite with piezoelectric performance. Since cement-based piezoelectric composite materials were proposed, the effect of the materials, content, and size of piezoelectric particles on its final properties have been investigated, as summarized in Table 3. It is clear to realize that the piezoelectric properties of Portland cement-based composites usually increase with the increase of lead-bearing materials content at the micro- and nano-level. The effect of size (ranging from 0.0236 to 620μm) on piezoelectric performance has also been investigated. Huang et al. [82] evaluated the size effect on piezoelectric properties and considered that the large particle (>100 μm) can insufficiently affect the *d*_33_ and *g*_33_. Li et al. [97] believed that the nano-powders with good crystallinity and distribution in the cement matrix can prepare the composite with higher performance. Hunpratub et al. [74] investigated the influence of BCTZO size and content and indicated that the particles with a smaller size can obtain higher performance due to the better connection. Kantakam et al. [78] characterized the dielectric properties of the geopolymer-based composites, revealing that the increase of the ceramic particle content will lead to the decrease of$\;\varepsilon_{r}$, but $\varepsilon_{r}$ is always greater than 1000, which may attribute to the influence of a particular matrix. Apparently, the content and size of ceramic particles can dramatically affect the density [70,98], which provides an approach to adjust the acoustic impedance. The particle shape has also been considered. Jaitanong et al. [91] investigated the interface and illustrated that the ceramic particles with angular shapes contribute to better bonding properties. The influence of the size, content, and shape on the final piezoelectric properties has been demonstrated. However, there are many other factors related to the material contributing to the final performance, such as bonding with cement matrix, which is difficult to summarize into a single problem, and the larger particles may lead to the loose structure [98,99]. With the increase of the ceramic content, a lower cement content may result in poor bonding behavior between the function phase and matrix. Therefore, the appropriate piezoelectric particle should be considered further based on the total material system. In summary, the larger particles and higher content are beneficial to improve piezoelectric performance, while the oversize particles will lead to the lower performance improvement efficiency, and the excessive content will cause bonding defects.

### 4.2. Matrix of CPCs

Polymers were usually employed as the matrix of piezoelectric composites [101,102] for the sensing element. Some natural defects exist in polymer/piezoelectric composite, such as the compatibility issue between the polymer and ceramics due to the significant difference in their composition and aging. The compatibility between the host material and the inbuilt sensing element has been another issue, attracting increasing attention. Cement, as an inorganic material, with excellent durability, can be mixed with piezoelectric particles directly, thereby obtaining inorganic composites. Cement-based piezoelectric composite can match the acoustic impedance and mechanical properties of concrete, more applicable to concrete-based infrastructure. However, the complex microstructure of the cement caused by the hydration products, porosity, long-term physical changes, and chemical reaction leads to an adverse impact on CPCs in the fabrication process, polarization process, and final performance.

Additionally, the occurrence of ITZ in the composite with weakened bonding behavior due to the difference in the growth rate of hydration products (e.g., CSH gel and calcium hydroxide crystal) is also a challenge. Different cement types have different chemical processes and durations, which requires selecting the curing condition and duration based on cement properties in the fabrication process. It is a delicate issue of CPCs between the cement matrix and piezoelectric materials that the higher temperature can help obtain the better piezoelectric properties but destroy the cement matrix due to chemically bound water loss. In the polarization process, the decomposition of chemically bound water caused by the high temperature and the product structure changes due to the extremely high voltage can increase the volume content of porosity and lead to the matrix strength loss. One issue for CPCs is that the matrix fabricated by various cement leads to the distinction in its final piezoelectric performance [47]. Each type of cement consists of a different component, resulting in the hydration products presenting specific forms, thereby affecting the final performance. Another issue is that specific types of cement, which with excellent properties including but not limited to high conductivity, high strength, and low porosity, such as geopolymer, can be an alternative matrix material. Due to the component difference in different cement types, further studies about the influence of cement type on the CPCs should be carried out. Table 1 and Table 2 show that the geopolymer-based composites show a higher dielectric constant and the inverse trend of piezoelectric performance by mixing higher-volume fractions of ceramic particles, which is an excellent inspiration to develop CPCs with various cementitious materials as a matrix.

Up to now, Portland, sulphoaluminate, and geopolymer cement have been employed to fabricate the composite. Additionally, to mitigate the mismatching, Zhao et al. [57] prepared the cement-sand-based piezoelectric composite, believing that it can improve mechanical strength, despite leading to a low-efficiency polarization process. This study also shows the difference in performance brought from the matrix. Although some studies about the influence of the matrix properties [48,78] have been carried out, it is still difficult to control the effect of the matrix on the performance of composite materials due to the complexity of the hydration characteristics of cement materials and the sensitivity of bound water to high temperatures. Concrete materials can self-heal [103] due to secondary hydration, carbonation, etc. This ability may contribute to the performance variation with time. Aging, related to the degradation with age after polarization, is caused by the releasing behavior of internal stress and redivision of ferroelectric domains, which is one of the major factors determining the stability of the piezoelectric performance. Dong et al. [37] found that the *d*_33_ of polymer-based composites after aging show a decreasing trend, while the *d*_33_ of CPCs show an increasing trend. Wang et al. [52] believed that the *d*_33_ could increase after polarization until the hydration process is completed. Considering the adverse impact of the free water on the polarization process, Pan et al. [83] pointed out that the *d*_33_ of CPCs with the high-temperature treatment can reach stable values earlier. However, the excessively high-temperature treatment can easily change the chemically bound water to free water, resulting in the matrix defects. It illustrates that the influence of water on the polarization may be more significant than that of defects. Additionally, some studies demonstrate that the direct piezoelectric effect can be characterized in hardening cement paste [104] and geopolymeric mortar [105] due to the presence of water, but there is no evidence to prove the contribution of the matrix to the piezoelectric effect. Although the composite’s aging shows a positive impact on the piezoelectric performance, it may lead to the uncontrolled final performance.

Currently, several issues related to the matrix have been investigated, as summarized in Table 4, which can be classified into (a) ITZ, (b) mechanical properties, (c) leakage current, (d) fabrication period, and (e) performance variance. The matrix properties mainly determine the ITZ, adequate curing promotes the cement to obtain a higher hydration degree and the admixture contributes to a denser matrix, thereby optimizing the ITZ and reducing defects. Compared with the ceramics and cement, the elastic modulus of cement is much lower. Adding admixture with higher elastic modulus is an excellent approach to modify the mechanical properties. A complex issue is that the polarization process can endow the ceramic with piezoelectric properties while causing damage to the matrix. Meanwhile, the ions and pores in the matrix may result in the occurrence of leakage current. Although the higher temperature and stronger electric field can destroy the matrix, it can help obtain better piezoelectric performance. Cheng et al. [95] considered the appropriate polarization temperature based on the decomposition temperature of hydrated cement and characterized the performance in various time and electric fields; Pan et al. [60] considered the moisture effect on CPCs and performance per-heat treatment, thereby obtaining lower dielectric losses; Wittinanon et al. [38] modified the microstructure of matrix by mixing PVDF, and the leakage current reduced dramatically. Although the variety of piezoelectric performance caused by long-term hydration of cement has been realized and promotes the utilization of quick-setting cement, fewer studies illustrate its advantage. Due to aging and the interference of piezoelectric ceramics on the cement hydration [77], the final performance is difficult to predict.

### 4.3. Admixture for CPCs

Generally, obtaining a denser matrix and improving the conductivity and mechanical properties are the basic ideas for selecting admixture. The effect of admixture on the piezoelectric performance of CPCs is summarized in Table 5. The lower piezoelectric performance is a common issue in piezoelectric composites, and the improvement of *d*_33_ with admixture is the most noteworthy. The electromechanical coupling coefficient (*K_t_* and *K_p_*) and dielectric constant (*ε_r_*) were also characterized. Many admixtures have been studied, showing the complex relationship between the piezoelectric performance and admixtures content. The excess electric conductivity will reduce the piezoelectric activities [55,56]. Meanwhile, the piezoelectric performance optimization by pozzolanic materials after a long period was also observed. Currently, the admixture mainly includes carbon materials, pozzolanic materials, and polymer. According to Table 5, these materials could improve the final performance efficiently, while excessive additives would cause performance degradation. The distinction of admixtures in physical and chemical properties, such as size, shape, and component, can lead to a significantly different performance. With the in-depth understanding of this composite, the research on the acoustic impedance and mechanical properties of admixture has gradually been paid more attention. There is a negative effect on piezoelectric performance caused by the distinction of elastic modulus between cement and piezoelectric ceramic particles. Concerning the efficiency of stress transfer between the matrix and ceramic particles, basalt fiber [43] is employed to improve the matrix’s elastic modulus and mitigate the stress buffer, thereby increasing the mechanical–electrical response. Admixture usually can positively impact the matrix compactness, thereby affecting the overall mechanical properties and acoustic impedance. Wittinanon et al. [38,39] revealed that PVDF could simultaneously control the piezoelectric properties, mechanical properties, and microstructure.

### 4.4. Further Study on Materials

The material selection can fundamentally determine the fabrication process and dramatically affect the final performance. Selecting materials should consider factors including the material availability and uniformity, piezoelectric performance, and convenient fabrication. In terms of piezoelectric materials, PZT has been widely used as a common material for CPCs. Lead-free piezoelectric materials are a promising candidate for the function phase of composite. The chemical reaction between piezoelectric ceramic and cement [96] can enhance the interface via chemical bonding. A case study on polymer/piezoelectric ceramic by Zheng et al. [108] has demonstrated the efficiency in improving piezoelectric performance by enhancing interface bonding with chemical reaction. Therefore, an optimization strategy is to modify the surface of piezoelectric ceramic particles, enhancing bonding. As the matrix phase, cement can also dramatically affect the piezoelectric properties. Scholars have employed some eco-friendly cementitious materials (e.g., geopolymers, magnesium phosphate cement), showing good durability and mechanical properties and controllable setting-time, which can be the potential matrix. Admixture is usually regarded as the material phase with an adjustment effect to optimize the mechanical and piezoelectric properties. The chemical reaction between the piezoelectric ceramic and cement matrix may occur in their ITZ, and the stress buffer [43] in ITZ leads to a low local piezoelectric strain factor. Further studies should investigate the mechanical properties and chemical bond of the composite, especially in the ITZ.

## 5. Fabrication Process of CPCs

### 5.1. General Fabrication Process

The fabrication process of CPCs is related to shaping the composite, modifying the microstructure, and reactivating the electrical response. The process mainly includes: (i) mixing, (ii) forming, (iii) curing, (iv) polishing, (v) coating, (vi) drying (heat treatment), and (vii) electrode forming and polarization. The (i)~(vi) steps can be called the preparation process, while the (vii) step is regarded as the polarization process. The preparation process relates to the shape of materials; the polarization process is in terms of functionalization. In polarization, the extreme temperature and electrical treatment process can cause certain matrix defects. In turn, the original defects of CPCs can adversely impact the electrical response efficiency, even resulting in a composite scrap [95]. Preparation parameters have a profound impact on CPCs, and although those parameters can be considered based on the properties of the materials, there are still some issues that can adversely impact the final performance.

### 5.2. Preparation Process

During the preparation process, the forming, curing, and drying (heat treatment), show a significant influence on the mechanical properties and later functionalization. Considering the defect caused by the conventional grouting molding method and the natural pores of cement materials, the compression molding method was employed to modify the microstructure, and later the influence of the forming pressure on piezoelectric properties was studied [59]. Due to the improvement of piezoelectric properties, the compression molding method became a major molding method for fabricating CPCs, deeming that the denser matrix can be obtained. However, excessive pressure will lead to internal micro-cracks, thereby lowering the density. Subsequently, the curing process for cement hydration is essential, which could improve the integrity of CPCs due to the chemical bonding and chemical reaction between the cement matrix and ceramic [96]. The free water will reduce during the drying process [60], improving the polarization efficiency.

### 5.3. Functionalization Process

Piezoelectric ceramics for CPCs are the artificial polycrystalline materials, with electric domains arranged in arbitrary orientation inside the micron-level grains, displaying no piezoelectricity macroscopically before polarization. To obtain the macroscopic piezoelectricity, the higher temperature, mechanical stress, or electric field can be employed to change the domain structure (also called dipole line-up), reversing the 180° domain and rotating the 90° domain. Generally, an electric field combined with increased temperature (below the Curie temperature) is regarded as a suitable approach to help piezoelectric ceramics generate the macroscopic polar axis and piezoelectricity easily, even though it will lead to the internal crack. For CPCs, the polarization is the same as pure piezoelectric ceramics, but the negative impacts are more complex due to the cement matrix and additives. Electric field, temperature, and polarization time are the major factors for the piezoelectric performance, but firstly, the electric field is the essential condition to be considered.

The local electric field (*E*_1_) on the piezoelectric particles of CPCs and the average electric field (*E_2_*) on the matrix can be expressed as [81,102].

This is example 1 of an equation:

(29) $$E_{1}=\frac{3\varepsilon_{2}}{\varepsilon_{1}+2\varepsilon_{2}}E_{0},$$

(30) $$E_{2}=E_{0},$$

where $\varepsilon_{1}$ and $\varepsilon_{2}$ are the dielectric permittivity of piezoelectric particles and cement matrix, respectively; *E*_0_ is the external electric field.

**Figure 6:**
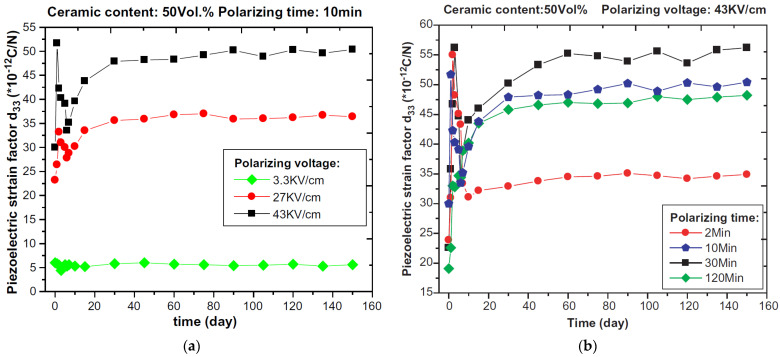
(**a**) The effect of polarizing voltage on *d*_33_ for 0-3 type cement based PZT composites. Reprinted with permission from ref. [61]. Copyright 2020 Elsevier. (**b**) The effect of polarizing duration on *d*_33_ for 0-3 type cement-based PZT composites. Reprinted with permission from ref. [61]. Copyright 2020 Elsevier.

Additionally, based on the molecular level mechanism, Li et al. [109] illustrated the relationship between the dipole domains of piezoelectric ceramic particles and macroscopical parameters of CPCs. This mechanism reveals the polarization efficiency, which can also help understand the relationship between the polarization and macroscopic piezoelectricity. The description of the mechanism is [109]:

(31) $$P=\left(\kappa^{\prime }-1\right)\varepsilon_{0}E=N\alpha E^{\prime },$$

where *P* is the polarization, equal to the dipole moment of each unit volume of the piezoelectric ceramics; $\kappa^{\prime }$ is the relative dielectric constant of piezoelectric ceramics; $\varepsilon_{0}$ is the permittivity of vacuum; N is the number of the contribution elementary particles per unit volume; α is the polarizability; $E^{\prime }$ is the local electric field; *E* is the applied electric field. From Equation (29), *E*_1_ depends on the dielectric permittivity of the piezoelectric particle and cement matrix. Equation (30) reveals the strong electric field applied to the matrix. The much higher $\varepsilon_{1}$ and lower $\varepsilon_{2}$ can lead to the smaller *E*_1_ during polarization. Additionally, Equation (31) describes the direct relationship between the applied electric field and macroscopic piezoelectricity, while *P* describes the macroscopic piezoelectricity from the molecular level, representing the charge density on the surface. From Table 2, it is clear that the $\varepsilon_{1}$ that have been applied in CPCs range from about 1000 to 3600, while $\varepsilon_{2}$ is about 40 to 530, which leads to the lower *d*_33_, and the appropriately high electric field density can help achieved a higher piezoelectric performance (Figure 6a). The *d*_33_ is directly related to the electric field, and the higher electric field intensity can help obtain a higher *d*_33_. However, the migration of weakly conductive ions (OH^−^, Ca^2+^, etc.) [95] in the cement matrix under the applied electric field will cause the decrease of breakdown voltage, which possibly leads to polarization failure. The excessive polarization time can also lead to inefficient polarization even though the appropriately high electric field was carried out. Dong et al. [61] characterized the negative effect of long polarization time on the piezoelectric performance (Figure 6b) and considered that a part of saturated piezoelectric particles has been broken down. Figure 6 also indicates the improvement of *d*_33_ with age, which seems to be contrary to the characteristics of piezoelectric ceramics. Chaipanich et al. [63] believed that the remnant polarization of the piezoelectric particles will reduce with time because the 90° ferroelectrics domains gradually become disordered, but the hydration of cement after polarization can densify the matrix, thereby improving the *d*_33_.

**Figure 7:**
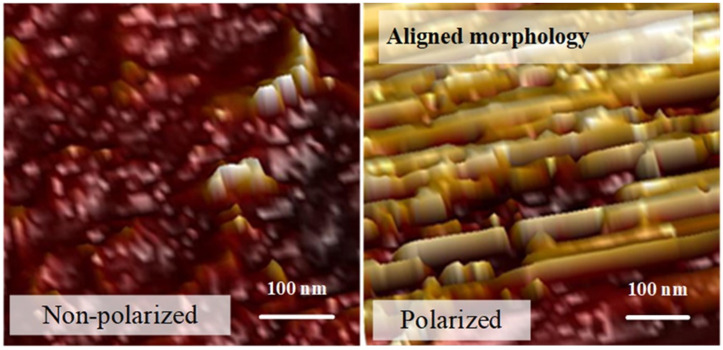
Atomic force microscopy (AFM) for non-polarized and polarized cement. Reprinted with permission from ref. [79]. Copyright 2019 Elsevier.

The microstructure of CPCs will change under the applied electric field and higher temperature during the polarization process. Currently, the polarization temperature of CPCs with lead-based piezoelectric ceramic is about 80~160 °C, which could lead to the instability of crystal water in hydration products (CSH gel, Al_2_O_3_·3H_2_O, etc.). Furthermore, the occurrence of chemical reactions and water loss would result in additional internal defects. Under the applied electric field, weakly conductive ions (OH^−^, Ca^2+^, etc.) [95] in the matrix will accumulate at defects, pores, and ITZ, thereby generating an opposing electric field and resulting in polarization suppression [59]. Chaipanich et al. [71] characterized the ferroelectric hysteresis of cement-based BZT composites at room temperature, and the polarization-electric field loops (P-E loops) show a lossy feature, which was also inferred to be the result of weakly conductive ion migration. The electric field can also produce a polarization effect in the cement matrix and change its electric dipoles alignments (see Figure 7) because calcium-silicate-hydrate (CSH) can obtain a better uniform nano-scale morphology and then reduce the ion transportability, thereby enhancing the output voltage. However, the contribution of electric treatment to the piezoelectric properties of CPCs is limited, even leading to some mechanical issues. A strong electric field and the ions in the pores could lead to the directional structure, inspiring further study on the matrix’s microstructure after polarization.

The high temperature would mitigate the suppression effect, reducing the cement matrix’s resistivity, thereby improving the polarization efficiency. It could also enhance the electric domain rotation and more easily realize directional array under the applied electric field, thereby obtaining better piezoelectricity. Meanwhile, a higher temperature could reduce the electric domain’s obstruction in the turning process and make the polarization process much easier in a relatively lower voltage. However, the defects caused by the chemical reaction under a higher temperature would generate the loose matrix and increase ion transportability, which further led to the decrease of the matrix resistance and resulted in the CPCs breakdown in an applied electric field. Lead-free piezoelectric materials have been employed in the composites, performing the polarization at the temperature of 60~80 °C, which could reduce the matrix damage.

We summarized the performance and polarization parameters of CPCs with different piezoelectric ceramic types in Table 6. Due to the almost identical piezoelectric particle size and content, and cement type, the final performance and fabrication parameters are easy to be compared. The *d*_33_ of lead-bearing and lead-free materials (with about 425 μm mean particle size) are similar, while the temperature and electric field for lead-free materials is lower than that of lead-bearing materials. For lead-free materials, although the low Curie temperature and thermal stability are the disadvantages affecting the composite application in severe environments, the lower polarization temperature could reduce its effect on the matrix. When the temperature is over 80 °C, the bound water of Portland cement will be lost. A lower temperature can mitigate the damage and water loss, thereby improving the density of the composite.

Therefore, a suitable temperature and electric field can be considered as coupling conditions, essential for the piezoelectricity activation of CPCs. The fewer internal defects (pores, ITZ, etc.) could also significantly contribute to the polarization efficiency. The polarization process parameters should consider the effect of ions and pores in the matrix because it can result in the local piezoelectric performance difference. Pre-heating may be an appropriate approach to reduce the water in the matrix to address the interference issue between the temperature and electric field. The material type is also another factor contributing to the parameter selection, especially the ceramic type.

### 5.4. Future Study on Fabrication

According to different material characteristics, it is essential to select the reasonable forming pressure and curing mode and optimize the fabrication process based on the different material types. Concerning the significant effect of stable piezoelectric performance on the composite as a sensing element, the approach to accelerate the aging period is essential. After polarization, the further hydration of the cement matrix is the major reason for the increase of piezoelectric properties with age, which enlightens the post-processing exploitation to stabilize the piezoelectric property quickly. High-temperature curing could be a potential approach. There are some challenges in considering fabricating the composite with eco-friendly materials and a faster preparation process. A good advantage of lead-free barium zirconate titanate for the composite is low-temperature polarization. Like geopolymer, some new types of cement with good dielectric properties can be the promising matrix. Recently, 3D printing is one of the potential manufacturing approaches and has been employed in sensor design and manufacturing [110]. The application of additive manufacturing technology in polymer-based piezoelectric composites’ [108] structure design and preparation also proves the feasibility of the piezoelectric composites combined with additive manufacturing technology, attributed to its good flexibility, and easy processing and molding, showing a promising prospect in combining smart manufacturing technology with sensor fabrication. Song et al. [111] employed 3D printing to prepare novel aggregate-shape embedded piezoelectric sensors. Chen et al. [112] fabricated a focused ultrasonic array with BaTiO_3_ ceramics via 3D printing, showing a good advantage in the size and shape control of piezoelectric ceramics. Lejeune et al. [113] fabricated piezoelectric ceramic with micro size by 3D printing. Considering the importance of the shape and size of piezoelectric particles for CPCs, this technique can be an approach to support the composite with controllable piezoelectric performance.

## 6. Application of CPCs

### 6.1. Application of CPCs

The application of the cement-based piezoelectric composite using various techniques is listed in Table 7. Considering the excellent compatibility of CPCs with concrete structure, the composites were encapsulated as the sensing element, namely cement-based piezoelectric ceramic composite sensor, which has been employed to monitor several issues, including cracking, steel corrosion, deterioration, and the hydration process. Those studies cover the issues encountered in the life cycle of concrete-based infrastructure and the critical stage of concrete forming, thereby showing the prospects of CPCs in SHM. Lu et al. [40] fabricated the CPCs sensor and analyzed its detection ability via acoustic emission (AE). The results show that the embeddable sensor has a good detective performance due to the reduction of external interference and improved ability to detect the AE signals induced by the micro-cracks or dislocation. Another property of this composite is the broadband characteristic, which can help analyze the loading process via AE frequency content variation [29,114]. These advantages of the composite are also presented in the case studies showing in Table 7. Dynamic mechanical monitoring can also be carried out based on this composite. Dong et al. [66] monitored the mechanical–electrical response of the composite, reporting that the electrical output signal could reproduce the mechanical input signal with quite a complex waveform and frequency range without any visible distortion. It holds a good performance to the dynamic mechanical signals input directly in beam, frame, and transporting simulation tests and exhibits excellent application potential in civil engineering. Considering the durability and stability of the CPCs sensor, Wang et al. [115] investigated the location of the composite element in the sensor and characterized its mechanical–electrical responses in various conditions. The results showed that the sensor possessed excellent linear performance when the ratio of cement to epoxy resin was 3:1 and the sensing element was put in a position near the underside of the encapsulation material; the fatigue load and water had a negligible effect on its linearity and sensitivity, and in the intended temperature range (0~40 °C), the sensor showed good linearity, almost independent of temperature. Recently, based on the *d*_33_ (99 pC/N) improvement, Pan et al. [31] embedded the cement-based sensor in a mortar specimen to detect its compressive strength growth via the EMI technique and compared with a PZT sensor, reporting that a more sensitive distinction was observed in the electrical impedance before and after it was embedded. Moreover, its effective monitoring frequency is more easily obtained during the mortar strength monitoring, which is beneficial for evaluating the change in the material properties in SHM with higher accuracy.

### 6.2. Further Application

Generally, the SHM system is expected to operate stably and continuously under the service environment. A cement-based piezoelectric ceramic composite sensor with superior durability can be embedded in concrete, thereby mitigating environmental interference. Due to the broadband property and excellent durability, the CPCs sensing element is expected to achieve long-term and large-scale monitoring in infrastructure. Additionally, considering the many advantages of the cement-based piezoelectric composite, the integrated multifunctional application deserves to be investigated. Sustainability is the requirement for infrastructure. The energy-harvesting system has become a strategic object and is expected to be combined with piezoelectric materials. Based on the principle of electromechanical conversion, this composite could also be an energy harvester [79] that collects energy under stable operation and act as an energy source in the power-off state.

## 7. Conclusions

Structural health monitoring (SHM) is the essential item for infrastructure to guarantee the safety and responsibility of sustainability and economic efficiency. CPCs, durable materials with compatibility for concrete, potentially achieve the sustainable and compatible SHM, but some issues still exist in its materials design and fabrication process. The profound understanding of CPCs can inspire systemic design and fabrication and expand its application in concrete-based infrastructures. This paper performs a comprehensive literature review on cement-based piezoelectric composites, including theories and experiments analysis, materials, the preparation process, and application. The following conclusions are summarized:

### 7.1. In Terms of Theories, Experimental and Simulation Study

Theoretical research for piezoelectric materials can promote understanding the advantage and working process of cement-based piezoelectric composites. The two-phase model can also clearly describe the action process between the matrix and piezoelectric particles. The imperfect surface and mechanical properties mismatch between the matrix and function phase will significantly affect the piezoelectric performance. In terms of experimental methods, the microstructure and piezoelectric performance are the essential items in characterization. Investigation on the mechanical property has attracted increasing attention, attributed to the insufficient stress transfer between the matrix and piezoelectric ceramic particles. More mechanical properties parameters of CPCs after polarization need to be further investigated, which help conduct performance prediction by combining with the simulation.

### 7.2. In Terms of Materials

The factors considered for the three components are different. For ceramic: (i) content, (ii) particle size, (iii) shape, (iv) lead-based/lead-free ceramics, and (v) particle surface, can be considered to optimize the composite performance. For matrix, it is essential to consider the properties, including (i) component, (ii) hydration product/hydration rate, (iii) density, and (iv) dielectric properties. For admixture, the consideration of improving (i) conductivity, (ii) density, and (iii) mechanical performance of the composite should be carried out. Lead-based/lead-free piezoelectric ceramic can be the function phase for the composite. Innovative cement materials are becoming more readily available. More innovative cementitious materials are worth using as the matrix for CPCs, thereby promoting the performance breakthrough and assisting in the highly efficient preparation process. The admixtures could cooperate with cement to obtain a denser matrix and enhance the matrix conductivity, which deserves further research.

### 7.3. In Terms of Fabrication Process

The fabrication optimization can achieve based on the properties of CPCs, especially the cementitious materials, due to the conflicts between the cement matrix and piezoelectric materials. The attention on the matrix performance improvement is valuable. Porosity and moisture in the matrix are the major factors contributing to the polarization inefficiency and breakdown. The temperature should be mainly determined based on the matrix and ceramic type; polarization time can be considered according to the content and size of ceramic particles; the electric field should endow the ceramics with higher performance and the matrix from breakdown. The application of intelligent manufacturing in the sensor fabrication process is worthy of attention.

### 7.4. In Terms of Application

The embeddable cement-based piezoelectric sensor has been employed to monitor the process, including cracking, steel corrosion, hydration, and strength development. Compared with traditional piezoelectric sensors, the lower d_33_ still restrict the application of CPCs. Realizing the long-term and large-scale infrastructural health monitoring based on multi-functionalization is one of the strategies of this piezoelectric composite. The CPCs with the ability to harvest energy are a potential application to achieve self-power.

## Figures and Tables

**Table 1:** Physical properties of piezoelectric ceramic, cement paste and concrete.

Items	Materials		
	Piezoelectric Ceramic	Cement Paste	Plain Concrete
Density (10^3^ kg/m^3^)	4.64–7.6 [33,38,39]	2.0–2.2 [37]	2.4 [40]
Acoustic velocity (10^3^ m/s)	2.83–3.40 [40]	2.64–3.37 [37,40]	3.0–4.2 [37,40]
Acoustic impedance (MRayl)	21.2–30 [37]	3.5–8 [41]	6.9–10.4 [42]
Elasticity modulus (GPa)	50–75 [38,43]	10–20 [44]	19.0–48.6 [45]

**Table 2:** Piezoelectric properties of piezoelectric materials.

Items	Piezoelectric Materials			
	Lead-Based Piezoelectric Ceramic	Lead-Free Piezoelectric Ceramic	Lead-Bearing CPCs	Lead-Free CPCs
Piezoelectric strain factor *d*_33_ (10^−12^ C/N)	215~513 [79]	190~235 [38,39]	0.5~87 [45,80] 5 *~143 * [37,52,64]	4~61.5 [74,75]
Piezoelectric voltage factor *g*_33_ (10^−3^ Vm/N)	15.9~25 [79]	12.43~18.28 [38,39]	15~60 [34,81,82] 20 *~30 * [83]	7~33.59 [38,75]
Dielectric constant *ε_r_* (at 1 kHz)	1050~3643 [37,77]	1452~1726 [38,39]	43.5~536 [34,47,48] 1017.6~1834.2 [78] 280 *~890 * [60,64]	120~350 [38,39]
Thickness electromechanical coupling coefficient K_t_ (%)	40~67 [79]	-	9.47~28.19 [34,79] 13.16 *~13.53 * [53,60]	9~14 [67]
Acoustic impedance Z (10^6^ kg/m^2^ s)	21.2~36 [33,37]	25.2~34 [33,38]	~9.6 [32]	7.5~10.5 [67,70,75]

* Range of maximal value after aging in references.

**Table 3:** Properties of cement-based piezoelectric composites with different piezoelectric ceramic particles content and size.

Piezoelectric Particle			Cement	Properties	Findings	References
Content (%)	Size (μm)	Type				
35~70 vol. 35~65 vol.	6.45 (mean) 153.6 (mean)	PZT	Portland cement	*d*_33_/$\varepsilon_{r}$/*K_t_* ↑•⊙ *g*_33_↑•	■ 40~50 vol.% of ceramics is the optimal content for matching acoustic impedance of concrete.	Li et al., 2002 [34]
80 wt. 60~85 wt.	0~300 (mean) 166.5 (mean)	PZT/PMN	Sulphoaluminate cement	*d*_33_↑•⊙ $K_{p}$/*K_t_/g*_33_↑•	■ High polarization field (˂4kV/mm)/temperature (˂130 °C) and increased polarization time (˂45min) lead to the enhanced performance.	Huang et al., 2004 & 2005 [46,62]
18~50 vol.	153.6 (mean)	PZT	Portland cement	*d*_33_↑•	■ *d*_33_ increases with the curing time.	Li et al., 2005 [61]
80 wt.	1.45/2.34/9.03/ 27.17/35.97/43.46/ 58.43/68.51/107.08/294.07 (mean)	PLN	Cement	*d*_33_/*g*_33_/*K_t_/K_p_*↑•⊙ *Q_m_* ↓⊙	■ Larger PLN particles lead to the change of connectivity patterns; large particle (>100μm) can insufficiently affect the *d*_33_ and *g*_33_.	Huang et al., 2006 [82]
40~60 vol. 50 vol.	620 (median) 3.8/148.8/620 (median)	PZT	Portland cement	*d*_33_/$\varepsilon_{r}\;$↑•⊙ *tan δ*↓•⊙	■ Less contact area between the cement matrix and the PZT particles enhance the piezoelectric properties.	Chaipanich et al., 2007 [47,80]
50~70 vol.	300~600	PSZT	Portland cement	$\varepsilon_{r}$*/d*_33_/*K_t_* ↑• *tan δ*↓•	■ PZT modified by Sr and Sb can obtain better piezoelectric performance.	Chaipanich et al., 2009 [100]
35~80 vol.	0.0236 (mean)	PZT	Portland cement	*d*_33_/*K_t_*/$\varepsilon_{r}$↑•	■ PZT nano-powders with good crystallinity and high-quality network distribution in cement can obtain good piezoelectric performance.	Li et al., 2009 [97]
30~50 vol.	425 (median)	PZT	Geopolymer	*tan δ/*$\varepsilon_{r}$↓•	■ The $\varepsilon_{r}$ can reach 1017.6~1834.2, different from that of Portland and sulphoaluminate cement.	Kantakam et al., 2013 [78]
40~70 vol. 50 vol.	425 (median) 75/212/425 (median)	BZT	Portland cement	*tan δ*↓⊙ $\varepsilon_{r}$/*K_t_*/*d*_33_/Z_c/_*ρ_c_* ↑•⊙	■ Acoustic impedance and density increase with size and content increasing.	Potong et al., 2013 [70,71]
30~70 vol. 50 vol.	425 (median) 75/212/425 (median)	BT	Portland cement	*K_t/_Z_c_* ↑•⊙ P*↓⊙	■ Acoustic impedance increases with increasing particle size and content, while porosity decrease with larger particle.	Rianyoi et al., 2013 [67]
30~70 vol.	8.9/569.8 (mean)	BCTZO	Portland cement	$\varepsilon_{r}$↑•⊙ *d*_33_↓⊙, ↑• *tan δ* ↓•	■ A higher specific surface and lower interface space can lead to better connection in ceramic particles. ■ Low polarizations for smaller particles resulting in a lower dielectric constant.	Hunpratub et al., 2014 [74]
40~60 vol.	425 (median)	BT	Portland cement	P*↓• Z_c_ ↑• $\varepsilon_{r}$↑• *tan δ*↓•	■ Acoustic impedance increases while porosity decrease with increasing particle content.	Wittinanon et al., 2020 [38]

↑•: increase with the particle content increasing; ↑⊙: increase with the particle size increasing; ↑•⊙: increase with both the particle size and content increasing; ↓•: decrease with the particle content increasing; ↓⊙: decrease with the particle size increasing; ↓•⊙: decrease with both the particle size and content increasing; P*: porosity; Z_c_: acoustic impedance; *ρ_c_*: density.

**Table 4:** Effect of cementitious matrix on the performance of CPCs.

Issues	Factors	Approach	Findings	References
ITZ between cement matrix and ceramic particles	Higher hydration degree contributes to better ITZ Cement component and hydration products [52]	■ Better curing method and adequate curing time ■ Adding admixture	♦ Adequate during period lead to better ITZ and higher *d*_33_ [62]. ♦ Better microstructure is obtained [52].	Cheng et al., 2005 [62] Wang et al., 2012 [52]
Mechanical properties	Lower elastic modulus Lower mechanical strength Mismatching of mechanical properties between cement and ceramics	■ Adding admixture ■ Matrix with high elastic modulus	♦ Admixture with high modulus and the good bonding interface with cement enhances local stress [43], thereby improving piezoelectric performance. ♦ Adding sand as a part of matrix obtains higher mechanical strength [57].	Zhang et al., 2019 [43] Zhao et al., 2016 [57]
Leakage current/conductivity	Chemically bonding water decomposes at high temperature [46] Free water Pores Weakly conducting ions	■ Select the appropriate polarization temperature, field, and time ■ Per-heating treatment ■ Adding admixture	♦ Polarization temperature for sulphoaluminate cement should be lower than 130 °C, thereby reducing the loss of crystal water, and meanwhile 45min for polarization is efficient; it is easy to breakdown if polarization field is over 4 kV/mm [95]. ♦ Less free water leads to better piezoelectric performance [60]. ♦ PVDF fills the pore and reduces the leakage current [38].	Chen et al., 2004 [46] & 2006 [95] Pan et al., 2016 [60] Wittinanon et al., 2020 [38]
Fabrication period	Slow hydration process	■ Quick-setting cement as matrix	♦ Prepare CPCs with sulphoaluminate cement [59].	Huang et al., 2007 [59]
Performance variance	Aging Existence of ceramics hinder hydration [77]	■ Performance testing until stable	♦ The decrease of porosity leads to better stress transformation at first 90 days after polarization and enhance the *d*_33_ [52]. ♦ Continue hydration may lead to the increase of *d*_33_ [63]. ♦ Charge redistribution in CPCs partly attribute to the age and w/c [64]. ♦ Cement curing process contributes to unstable dipoles and piezoelectric phase constriction, resulting in aging fluctuations [77].	Wang et al., 2012 [52] Chaipanich et al., 2014 [63] Pan et al., 2020 [64] Santos et al., 2020 [77]

**Table 5:** Influence of admixture on the performance of CPCs.

Admixture	Content (%)	Matrix	Piezoelectric Ceramic	Properties	Consideration	Major Findings	References
Silica fume	5~10 wt.	Portland cement	PZT	*d_33_/*$\varepsilon_{r}\;$↑	Dense matrix	♦ Silica fumes make the matrix dense and improve piezoelectric performance.	Chaipanich et al., 2007 [51]
Carbon addition	1~2 vol.	Portland cement	PZT	$\varepsilon_{r}$/*tan δ* ↑	Continuous electric flux	♦ Carbon addition improves the dielectric behaviors.	Jaitanong et al., 2008 [54]
Carbon black	0~1 vol.	Sulphoaluminate cement	P(LN)ZT	*d*_33_*/g*_33_↑ *Kt/Qm =*	Conductivity	♦ Excessively high conductivity leads to poor piezoelectric performance.	Huang et al., 2009 [55]
Carbon black	0~1.7 vol.	White cement	PZT	*d*_33_/*K_t_/K_p_* ↑ $\varepsilon_{r}$/*tan δ*↗↘	Conductive phase	♦ Excess electric conductivity leads to lower piezoelectric activity.	Gong et al., 2009 [56]
Carbon nanotubes	0.1~1.3 vol.	Portland cement	PZT	*d*_33_*/g*_33_↗↘ $\varepsilon_{r}$/*tan δ* ↑	Conductive filler improves the polarization efficiency	♦ CNTs increase the polarization efficiency.	Gong et al., 2011 [81]
Silica-based material	10~20 wt.	Portland cement	PZT	*d*_33_↑*→*↑ $\varepsilon_{r}$↑	Enhanced phase Optimize the ITZ	♦ The *d*_33_ of silica-based composite (20 wt.%) cured at room temperature and dry atmosphere can reach 70pC/N after 38 days.	Wang et al., 2012 [52]
Slag Fly ash	10~50 wt.	Portland cement	PZT	*d_33_/*$\varepsilon_{r}$/*g_33_→*↓ *K_t_→=*	The performance changes with time Improve the strength of matrix	♦ The performance test results at a later period after polarization are more suitable as a representative value. ♦ Curing time shows little effect on the final performance.	Pan et al., 2014 [49]
Fly Ash	10~50 vol.	Portland cement	PZT	*d*_33_ ↗↘,*→*↑ *g*_33_↗↘,*→*↓ $\varepsilon_{r}$*→*↑	The performance changes with time	-	Pan et al., 2014 [106]
PVDF	1~20 vol.	Portland cement	PZT	*d_33/_g_33_/*$\varepsilon_{r}/$*tan δ* ↗↘	Being as a connecting third phase with desirable properties To promote polarization	♦ Adding PVDF to the composite significantly reduces polarization time.	Jaitanong et al., 2014 [107]
Kaolin	0~10 vol.	Portland cement	PZT	$\varepsilon_{r}$*→*↑,↑ *tan δ* ↑ *d_33_→*↑,↓ *Kt→=*, ↑*g*_33_=	Enhance ferroelectric behavior	♦ The composite mixed with kaolin reduces the porosity.	Pan et al., 2015 [53]
Carbon nanotubes (CNTs)	0~0.9 vol.	Portland cement and sand	PZT	*d_33_/*$\varepsilon_{r}$↗↘ *tan δ* ↑	Increase electrical conductivity for easier polarization	♦ Composite mixed with CNTs can be used to catch the stress changes.	Zhao et al., 2016 [57]
Carbon nanotubes	0~2 vol.	Portland cement	BNBK	*d*_33_/*g*_33_↗↘ $\varepsilon_{r}$/*tan δ* ↑	Increase electrical conductivity for easier polarization	♦ CNTs optimize the microstructure and improve the piezoelectric properties.	Potong et al., 2017 [76]
Graphene nanoplatelets (GNPs)	0~5 wt.	Silica fume blended Portland cement	PNZT	$\varepsilon_{r}$/*tan δ* ↑	Evaluate the effect of GNPs on morphological and electrical properties	♦ There is no particular concentration of GNPs, enhancing the dielectric behavior.	Jaitanong et al., 2018 [58]
PVDF	1~10 vol.	Portland cement	BNBT	$\varepsilon_{r}$/*tan δ*↓ *d*_33_↗↘	Efficient polarization	♦ Adding PVDF obtains a denser matrix.	Rianyoi et al., 2018 [75]
Fly ash	10~50 vol.	Portland cement	BZT	$\varepsilon_{r}$↓*tan δ* ↑ *d*_33_/D↓	Improve physical properties of matrix	♦ The density decreases and inadequate reaction of cement caused by excessive fly ash content led to the decrease of piezoelectric properties.	Chomyen et al., 2018 [50]
Basalt fibers (BF)	0~0.24 vol.	Portland cement	PZT	V*/g_33_↗↘ *tan δ*/$\varepsilon_{r}$↗↘ *d*_33_↓	Increase elastic modulus of matrix	♦ Local stress is significantly enhanced by BF with high modulus and good interface bonding with cement matrix.	Zhang et al., 2019 [43]
PVDF	0~7 vol.	Portland cement	BZT	$\varepsilon_{r}$↓ *d*_33_↗↘ P*/H_v_/H_k_/E↓ K_c_ ↑	Evaluate the effect of PVDF on microstructure, physical, mechanical and piezoelectric properties	♦ PVDF can optimize the physical, dielectric and piezoelectric properties by increasing densities.	Wittinanon et al., 2020 [39]
PVDF	0~7 vol.	Portland cement	BT	$\varepsilon_{r}$/*tan δ*↓ *g*_33_/*d*_33_ ↗↘ P*/Z_c/_A↓	Evaluate the effects of PVDF on the density, porosity, microstructure, acoustic impedance, dielectric and piezoelectric properties	♦ Adding PVDF can optimize the microstructure, control acoustic impedance, and reduce porosity and leakage current.	Wittinanon et al., 2020 [38]

↑: increase with the admixture content increasing; ↓: decrease with the admixture content increasing; =: slight fluctuation with the admixture content increasing; ↗↘: increased first and then decreased with the excess admixture content increasing; *→*↑: increase with time (at the same content of admixture); *→*↓: decrease with time (at the same content of admixture); →=: slight fluctuation with time (at the same content of admixture). P*: porosity; V*: output voltage; H_v_: Vickers hardness; H_k_: Knoop hardness; E: elastic modulus; K_c_: fracture toughness; Z_c_: acoustic impedance; A: leakage current; D: density.

**Table 6:** Performance and polarization parameters of CPCs with different ceramic type.

Piezoelectric Ceramics			Cement Type	Admixture	Fabrication					*ε_r_*	*d*_33_(pC/N)	*tan δ*	References
Ceramics Type	Particle Size (μm)	Content (vol. %)			Forming Pressure (MPa)	Curing Time/Temperature (day)/(°C)	Temperature (°C)	Electric Field (kV/mm)	Time (min)				
PZT	620	50	OPC	N	80	3/60	130	2	45	176	26	0.79	Chaipanich et al., 2007&2008 [47,80]
PZT	450	50	OPC	N	-	3/60	130	2	45	120	17	-	Chaipanich et al., 2007 [51]
BZT	425	50	OPC	N	-	3/60	50	1	45	350	14	0.87	Potong et al., 2013 [70,71]
BCTZO	569.8	50	OPC	N	146	3/60	R*	1	45	80.7	18	0.1	Hunpratub et al., 2014 [74]
BNBK	425	50	OPC	N	-	3/60	80	1.5	45	188	41	~0.7	Potong et al., 2017 [76]
BZT	425	50	OPC	N	-	3/60	60	0.5	45	1220	16.28	0.73	Chomyen et al., 2018 [50]
BNBT	425	50	OPC	N	-	3/60	60	0.5	45	~300	~26	~0.6	Rianyoi et al., 2018 [75]
BT	425	50	OPC	N	-	3/60	60	1	45	249	17	0.66	Wittinanon et al.,2020 [38]

R*: polarization in room temperature; N: without admixture; -: cannot obtain accurate value from literature.

**Table 7:** Application of cement-based piezoelectric ceramic composites.

Techniques	Objects	Achievements	References
Mechanical–electric response	Dynamic mechanical evaluation	♦ Small nonlinear piezoelectric effect with minimal impact. ♦ Excellent performance in characterizing dynamic signals. ♦ Excellent electrical output signal repeats the complex mechanical input signal.	Dong et al., 2011 [66]
	Sensor performance characterization	♦ Rapid response to load. ♦ Excellent load transmission relates to the position of CPCs in the sensor. ♦ Better sensitivity in higher temperature.	Wang et al., 2014 [115]
Acoustic emission technique	Investigate AE signals detect ability of the sensor Crack detection Corrosion process of reinforced concrete beam	♦ In-built cement-based sensor avoids the distortion of AE. ♦ Cement-based piezoelectric composite with broadband properties can efficiently monitor the concrete structure deterioration.	Lu et al., 2008 [40]; 2010 [116]; 2011 [117]; 2011 [29,114]; 2012 [118]; 2013 [65]; 2016 [119]
Ultrasonic technique	Hydration process	♦ The active acoustic monitoring method based on the in-built cement-based piezoelectric composite is efficient to monitor the growth of solid phases in concrete.	Lu et al., 2013 [30]; 2014 [120]
Electromechanical impedance (EMI)	Strength development of the mortar	♦ In-built cement-based piezoelectric composite has the capacity to monitor the strength evaluation. ♦ Cement-based piezoelectric composite is easy to find an effective monitoring frequency due to broader frequency bandwidth.	Pan et al., 2020 [31]

## Data Availability

The data presented in this study are available on request from the corresponding author.
